# Do Bodily Expressions Compete with Facial Expressions? Time Course of Integration of Emotional Signals from the Face and the Body

**DOI:** 10.1371/journal.pone.0066762

**Published:** 2013-07-23

**Authors:** Yuanyuan Gu, Xiaoqin Mai, Yue-jia Luo

**Affiliations:** 1 State Key Laboratory of Cognitive Neuroscience and Learning, Beijing Normal University, Beijing, China; 2 Department of Psychology, Renmin University of China, Beijing, China; 3 Institute of Affective and Social Neuroscience, Shenzhen University, Shenzhen, China; University of Rome, Italy

## Abstract

The decoding of social signals from nonverbal cues plays a vital role in the social interactions of socially gregarious animals such as humans. Because nonverbal emotional signals from the face and body are normally seen together, it is important to investigate the mechanism underlying the integration of emotional signals from these two sources. We conducted a study in which the time course of the integration of facial and bodily expressions was examined via analysis of event-related potentials (ERPs) while the focus of attention was manipulated. Distinctive integrating features were found during multiple stages of processing. In the first stage, threatening information from the body was extracted automatically and rapidly, as evidenced by enhanced P1 amplitudes when the subjects viewed compound face-body images with fearful bodies compared with happy bodies. In the second stage, incongruency between emotional information from the face and the body was detected and captured by N2. Incongruent compound images elicited larger N2s than did congruent compound images. The focus of attention modulated the third stage of integration. When the subjects' attention was focused on the face, images with congruent emotional signals elicited larger P3s than did images with incongruent signals, suggesting more sustained attention and elaboration of congruent emotional information extracted from the face and body. On the other hand, when the subjects' attention was focused on the body, images with fearful bodies elicited larger P3s than did images with happy bodies, indicating more sustained attention and elaboration of threatening information from the body during evaluative processes.

## Introduction

As a highly social species, it is of great significance for humans to comprehend and interpret others' emotions, intentions, and actions. Nonverbal cues, such as facial expressions, bodily posture and gesticulation, and vocal prosody constitute a rich source of social signals. The ability to decode these signals contributes to successful social interactions. Compared to facial and vocal cues, nonverbal body language has been under-researched and has received little attention until recently [1], [2]. Recent studies have shown that human brains respond to nonverbal signals from the face and body in a similar manner. Specifically, an event-related potential (ERP) study found that, like facial expressions, threatening emotional information from bodily expressions can be extracted rapidly. This rapid detection was evidenced by shortened P1 peak latencies when participants viewed fearful whole body actions relative to latencies observed when they viewed neutral actions [3]. In addition, an imaging study found increased activation in the visual cortex when participants were presented with emotional bodies compared to neutral bodies [4]. Additionally, imaging studies have shown that emotional body postures can be perceived implicitly in the absence of the visual cortex (e.g., [5]), indicating that both cortical and subcortical pathways are involved in the processing of emotional bodies, similar to the situation with the processing of emotional faces [6].

Given that faces and bodies are normally seen together, it is natural to question how the decoding of emotions from the face and body would interact with each other. Research on the integration of multiple emotional signals is not a new topic in the area of emotion perception. However, previous investigations in this area have been mostly concerned with the integration of emotional signals from faces and voices while participants were attending to faces [7], [8] or voices [9]. Using an oddball paradigm, de Gelder and colleague [7] found that face-voice pairs with a deviant facial expression elicited a negative ERP component showing the characteristics of the mismatch negativity (MMN), when participants were asked to attend to the faces. Pourtois et al. [8] observed an enhanced N1 during another oddball task when participants were presented with congruent face-voice pairs (angry voice, angry face) relative to when they were presented with incongruent pairs (angry voice, sad face). Pourtois and colleague [9] further investigated how happy and fearful facial expressions influence auditory event-related potentials (AEPs) evoked by concurrent presentation of bi-syllabic words spoken with happy and fearful intonations, when participants were asked to attend to the voices. They found that incongruent face-voice pairs elicited later P2bs than congruent pairs. These findings suggest that emotional signals from faces can affect the processing of concurrently presented emotional voices whether participants are attending to faces or voices.

More recently, researchers examining the integration of emotional signals from different sources have begun to pay attention to bodily expressions. In an audiovisual integration study, Jessen and Kotz [10] showed that emotional whole body stimuli, which contained both facial and bodily expressions can, affect the processing of emotional vocal stimuli. Similar to previous findings [8], the emotional whole body stimuli affected the early stage of emotional vocal stimuli processing, as evidenced by reduced N1s in the audiovisual condition relative to the audio-only condition, whether participants were judging which emotion the actor was expressing or judging the length of the stimulus. Audiovisual integration studies often employ separate analyses of visual event-related potentials (VEPs) time-locked to the onset of visual stimuli and AEPs time-locked to the onset of auditory stimuli, with auditory stimuli being presented several seconds after the onset of the visual stimuli to reduce cross-modal interference. Investigation of the integration of facial and bodily expressions, two important visual nonverbal cues, allows for an online assessment of two emotional signals with the same onset times. It remains to be determined whether two visual emotional signals will integrate with a pattern similar to that observed with auditory and visual stimuli.

Only a handful of studies have examined the integration of emotional signals from the face and body. Behavioral experiments have shown that the recognition of facial expressions is biased towards simultaneously perceived bodily expressions, indicating a systematic influence of bodily expressions on the processing of facial expressions [11]. An ERP study found that the integration occurred rapidly and automatically, as evidenced by larger P1 amplitudes when the face and body were expressing incongruent emotions relative to when they were expressing congruent emotions [12]. However, the time course for the integration of emotional signals from the face and the body remains unclear. An imaging study [13] found that the combination of a fearful face with a fearful tone of voice resulted in increased amygdala activation compared to the combination of a fearful face with a happy tone of voice, suggesting that cross-modal merging of emotional information is driven by an integration of congruent meaning. It remains to be determined whether integration of facial and bodily expressions occurs with a similar pattern as facial and vocal cues, that is, whether emotional information is merged by integration of congruent meaning as time unfolds. Thus, the first part of our study was designed to address this question.

Early investigations by Ekman and colleagues [14] found that the judgment of a person's emotional state by observers was more accurate when made based on the body rather than on the face in a deceptive condition in which the person shown in the video was lying about their feelings. While there are exceptionally keen observers who can perceive others' emotional states from micro facial expressions, bodily expressions seemed to be a more reliable resource for most people to detect others' true emotions. Nonetheless, it is still unknown whether this reliance on bodily expressions leads to disproportionate weight being assigned to bodily expressions when emotional signals from the face and body are both available, especially when attention is focused on the body. Earlier studies of integration have focused on investigating the influence of bodily expressions on the processing of emotional signals from other sources, such as the face or voice, by having emotional signals from other sources attended to and bodily expressions left task-irrelevant [11], [12]. A recent study investigated how emotional voices and bodily expressions affect each other when bodily expressions are masked as to be visually unnoticeable [15]. The results showed that when emotional voices were task-irrelevant and congruent with bodily expressions, the recognition of bodily expressions was improved, whereas when bodily expressions were task-irrelevant, the interpretation of emotional voices was biased towards simultaneously presented bodily expressions. Other neural studies on audiovisual integration, however, have suggested that the focus of attention can modulate the early integration of visual and auditory stimuli. For example, when Talsma, Doty and Woldorff [16] combined a rapid serial visual presentation (RSVP) letter stream with simultaneously presented visual and auditory objects, they observed differing patterns of integration of audiovisual stimuli depending on whether the subjects' attention was directed at multisensory stimuli, (i.e., both visual and auditory objects), unisensory visual or auditory stimuli, or the RSVP letters. In the audiovisual attention condition (i.e., when both visual and auditory objects were attended to), the amplitudes of the early P50 components elicited by multisensory stimuli were larger than those elicited by the sum of unisensory visual and auditory stimuli. In the RSVP attention condition (i.e., when both visual and auditory objects were unattended), this effect was reversed, with multisensory stimuli evoking smaller P50s than the sum of unisensory stimuli. In the visual attention condition (i.e., when attention was directed to visual objects), an enhanced late frontal negativity was observed in ERPs elicited by multisensory stimuli relative to those elicited by summated unisensory stimuli. Taken together, the aforementioned studies of integration of signals from different sources suggest that the integration can occur when participants are attending to either one of the signals and that the integration pattern can sometimes be modulated by the focus of attention, revealed by distinct neural activities. Thus, the second part of our study was designed to test whether the focus of attention can modulate the pattern of integration of facial and bodily expressions.

An ERP analysis is well suited for examining the time course of the integration of facial and bodily expressions. The high temporal resolution of ERPs allows for online assessment of multiple information-processing operations after stimulus onset. ERPs have been a historically important method for studying psychological processes and individual ERP components have been associated with distinct information-processing operations, including aspects of attention. For example, P1 has served as an index of attention allocation during the early coarse processing stage. As more attention is allocated, P1 amplitude increases [17]. Meanwhile, the N200 component is sensitive to conflict detection [18], [19], and the P300 component, which occurs later in information processing, has been interpreted as a reflection of sustained selective attention directed at motivationally relevant input and is often used to observe more elaborate and evaluative processes [20].

The primary goal of the present study was to examine the temporal characteristics of the integration of facial and bodily expressions utilizing ERP technology. ERPs were recorded while participants watched face-body compound stimuli on a computer screen. Specifically, in Part 1, participants were asked to judge the facial expressions and ignore the body. In Part 2, participants were asked to judge the bodily expressions and ignore the face. We expected to observe an integration of the two emotional signals during the early stages of processing in both parts of the study. We also expected to obtain evidence of integration driven by the merging of congruent emotional meaning during the later stages of processing. If facial expressions affect the processing of bodily expressions in a manner similar to the way bodily expression affect the processing of facial expressions, then a similar integration pattern should be observed in the two parts of the study. If not, then distinct merging patterns should be observed.

## Part 1 Methods

The aim of Part 1 was to test the time course of integration of facial and bodily expressions when attention was focused on the face. The participants were asked to judge the facial expression presented in each face-body compound image as quickly and accurately as possible. A red fixation point was marked on the stimulus faces to facilitate the visual focus of the participants. The participants were instructed to focus entirely on the stimulus face and ignore the body.

### Ethics Statement

This study and the recruitment of participants were approved by the Beijing Normal University Institutional Review Board (IRB). Written informed consent forms were collected from all participants.

### Participants

Eighteen healthy right-handed undergraduates from Beijing Forestry University, aged between 18 and 25 years old (21.8±2.2 years; 9 females) participated in Part 1 of the study. Data from three participants were excluded because of excessive artifacts. Statistical analyses were then performed on the remaining 15 participants, which included 8 females and 7 males. All participants had normal or corrected-to-normal vision and had no history of affective disorders.

### Materials

To create face-body compound stimuli, 30 gray-scale face images (15 happy and 15 fearful face images) of 15 individuals (7 male, 8 female) were selected from the Chinese Affective Face Picture System (CAFPS) [21]. For body images, photos were taken from 29 actors (14 male, 15 female) expressing six different bodily expressions (happiness, fear, anger, sadness, surprise, and disgust). All actors were photographed against a white background in a studio. These photographs were then used in a validation study. In the validation, the images of bodily expressions were presented one at a time on a computer screen; each image was presented for 6000 ms with a 4000-ms inter-stimulus interval. Participants (N = 25) were instructed to judge the emotion expressed by the body in a forced-choice procedure, in which they chose one of the six emotions as quickly and as accurately as possible by pressing a key on the keyboard. They were also asked to rate the arousal level that the person in the image was experiencing on a 7-point scale, with 1 being “not aroused at all” and 7 being “extremely aroused”. The mean values of accuracy for images with different emotions were 57.69±6.18% (anger), 50.0±3.62% (disgust), 73.63±6.45% (fear), 73.07±3.51% (happiness), 72.0±6.29% (sadness), 56.52±7.71% (surprise). The means of arousal for the images were 3.95±0.81 (anger), 2.82±0.41 (disgust), 4.17±0.44 (fear), 4.48±0.47 (happiness), 1.43±0.41 (sadness), 3.23±0.47 (surprise).

**Figure 1:**
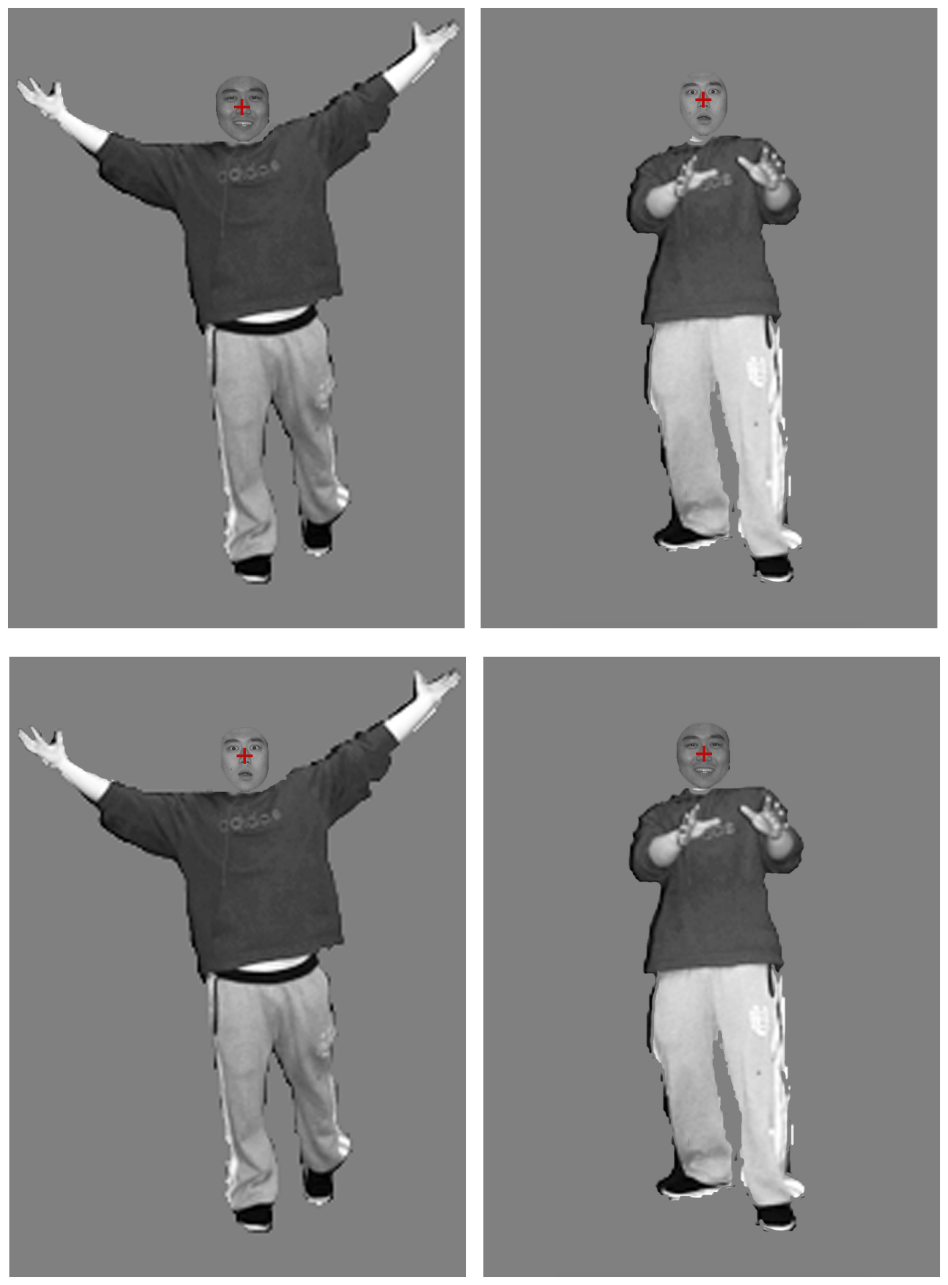
Examples of the Four Different Categories of face-body compound stimuli used in Experiment 1.

Thirty gray-scale body images (15 happy and 15 fearful body images) of 15 individuals (7 male, 8 female) were then randomly selected from the validated photos with recognition accuracy above 70%. The means of accuracy and arousal for the selected photos were 74.51±3.26% and 4.28±0.45, respectively. The happy and fearful body images were compounded with the faces using Photoshop software. A total of 60 compound images, 15 for each of four types, were then made by combining faces and bodies of the same gender in realistic proportions (face: body≈1: 7). A red fixation point was marked on the face. The four types, namely HappyFace-HappyBody, HappyFace-FearfulBody, FearfulFace-FearfulBody, and FearfulFace-HappyBody, were divided into a congruent condition in which facial expressions matched bodily expressions and an incongruent condition in which they did not. Examples of the stimuli used in Part 1 are shown in Figure 1.

### Procedure

During electroencephalogram (EEG) recordings, subjects were seated in a relaxed position on a comfortable chair in a dimly lit and electrically isolated room with their eyes at a distance of 80 cm from the screen. The size of the face-body compound stimuli picture on the screen was approximately 7×9.5 cm. The 60 compound images were presented six times, generating 360 trials in total. Part 1 was divided into four counterbalanced blocks, each of which included 90 trials. In each block, the images were presented in a pseudo-random order, and the same stimulus was shown no more than three times successively. Each trial started with a 200 ms black fixation point, which was located in the upper 33% of the screen at approximately the same height as the red fixation point on the face. After a blank screen randomized from 100 ms to 300 ms, the stimuli were then presented for 200 ms, followed by another black screen lasting for 1000 ms before the next trial. The participant's task was to decide the emotion expressed on the face as quickly and accurately as possible by pressing F or J on the keyboard. The key for happiness or fear was counterbalanced among the participants. The participants were asked to keep their eyes fixed on the face during this part of the study. Before the real trials, all participants went through a training session to get familiar with the procedure.

### ERP Recording

The EEG was recorded from 64 scalp sites using Ag/AgCl electrodes mounted in an elastic cap (Neuroscan Inc.), with reference to the left mastoid. The vertical electrooculogram (VEOG) and horizontal electrooculogram (HEOG) were recorded with two pairs of electrodes, one placed above and below the left eye, and another 10 mm from the outer canthi of both eyes. Interelectrode impedance was maintained below 5 kΩ. Signals were amplified with a 0.05–100 Hz bandpass and continuously sampled at 500 Hz/channel for off-line analysis.

### ERP Analysis

All EEG signals were re-referenced off-line to the average of the left and right mastoids [22]. The EEG data were digitally filtered with a 30 Hz low-pass and were epoched into periods of 1000 ms, each of which included a 200 ms pre-stimulus baseline. Ocular artifacts were removed from the EEG signal using a regression procedure implemented in Neuroscan software [23]. Trials with various artifacts were excluded, where peak-to-peak deflection exceeded ± 75 µV. The ERPs were then averaged separately for each type of stimulus.

The target ERPs were averaged separately from the time point of their onsets. For the P1 and N2 components, the peak amplitudes and latencies were measured. The time windows for the P1 and N2 components were 70–120 ms and 200–250 ms respectively. For the P3 component, mean amplitudes were measured in the time window of 350–700 ms after stimulus onset. The P1 component was analyzed at three occipital electrode sites: Oz, O1 and O2. The N2 component was analyzed at six electrode sites: Fz, FCz, F1, F2, FC1 and FC2. The P3 component was measured at three posterior electrode sites: Pz, P1 and P2. Repeated-measures analyses of variances (ANOVAs) were applied to different components in the ERP data analysis. The factors were Bodily expression (happiness *vs.* fear), Congruence (congruent *vs.* incongruent) and Electrode site. The Greenhouse-Geisser epsilon correction was applied to adjust the degrees of freedom of the F-ratios. Post hoc analyses were conducted to explore interaction effects.

## Part 1 Results

### Behavioral data

Two-way repeated measures ANOVAs of bodily expression (happiness *vs.* fear) by congruence (congruent *vs.* incongruent) were employed to analyze reaction times (RTs) and accuracy. A main effect of congruence was found for both accuracy [*F* (1, 14) = 32.73, *p*<.001] and RTs [*F* (1, 14) = 61.32, *p*<.001] in the task of judging facial expressions. Participants made significantly better (*M* = 94±3%) and faster (*M* = 560±53 ms) decisions when the faces were accompanied by bodies with congruent expressions than when they were accompanied by bodies with incongruent expressions (accuracy: *M* = 85±6%; RT: *M* = 596±52 ms). No other significant main effects or interactions were found.

### ERP data

#### P1 component

**Figure 2:**
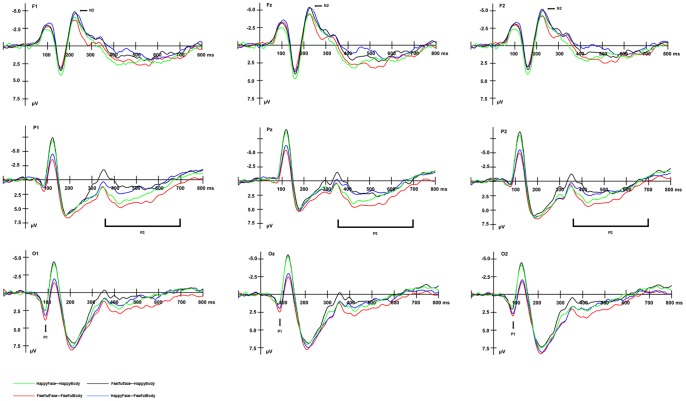
Group average ERPs for the four different compound stimuli at indicated electrode sites in Exerperiment1.

The repeated measures ANOVA revealed a main effect of bodily expression [*F* (1, 14) = 25.91, *p*<.001] for the amplitude of the P1 component at all three occipital sites. As shown in Figure 2, P1 was larger in amplitude for the compound stimuli with fearful body images (*M* = 2.92 µV) than happy body images (*M* = 1.94 µV) at occipital sites. There was also a main effect of electrode site [*F* (2, 28) = 8.11, *p*<.01], but post hoc tests failed to reveal significant differences between the three electrodes. No other main effect or interactions were significant.

To assess the effect of facial expression on the P1 component, another repeated measures ANOVA was applied to the data, with the factors being facial expression (happiness vs. fear), congruence (congruent vs. incongruent) and electrode site. The main effect of facial expression was not found to be significant. A main effect of electrode site [F (2, 28) = 8.11, *p*<.01] was significant, but post hoc tests did not reveal significant differences between the three electrodes. The interaction of facial expression and congruence was significant [F (1, 14) = 25.91, *p*<.001]. Post hoc tests revealed that when facial expression was fearful, congruent images elicited larger P1s than incongruent images (*p*<.01), whereas when facial expression was happy, congruent images elicited smaller P1s than incongruent images (*p*<.05).

#### N2 component

A main effect of congruence was found for the amplitude of the N2 component at anterior electrodes [*F* (1, 14) = 15.83, *p*<.001] (Figure 2). The N2 components elicited by compound images with incongruent expressions had greater amplitudes (*M* = −5.18 µV) than those elicited by images with congruent expressions (*M* = −4.29 µV). Although a main effect of electrode site was also found [*F* (5, 70) = 6.72, *p*<.021], post hoc tests failed to reveal significant differences between the six electrodes. No other significant effects were found.

#### P3 component

A main effect of congruence was also found [*F* (1, 14) = 17.16, *p*<.001] for the mean amplitude of the P3 component (Figure 2). The faces accompanied by bodies with congruent expressions (*M* = 2.54 µV) elicited a more positive deflection in the averaged waveforms than those accompanied by bodies with incongruent expressions (*M* = 1.12 µV). No other significant effects were found.

## Part 1 Discussion

Part 1 of the study demonstrated the time course of integrating emotional signals from the face and body when participants were instructed explicitly to focus on the face. First, the results showed that the threatening information conveyed by the body could be extracted rapidly in the early coarse processing stage, as evidenced by enhanced P1 amplitudes when participants viewed compound images with fearful bodies compared with those elicited by images with happy bodies. This finding is consistent with the results of a previous study, which also demonstrated that negative emotional information from the body was captured during this early stage of processing [12]. No such effect on P1 amplitudes was observed when the participants were viewing compound images with fearful versus happy faces. Second, the data also provided evidence for detection of incongruent emotional information from the face and body in the form of enhanced N2 amplitudes in the incongruent condition compared to the congruent condition, which indicated that there was indeed integration of emotional information from the two sources. Third, the data also showed that later evaluative processes may be driven by congruent emotional meanings of the two sources by way of more pronounced P3 waveforms for compound images with congruent emotional signals relative to images with incongruent signals.

## Part 2 Methods

The goal of Part 2 was to investigate the time course of the integration of facial and bodily expressions when attention was focused on the body. The participants were instructed to judge the bodily expressions as quickly and as accurately as possible. A red fixation point was marked on the center of the chest to direct the participants' focus. The participants were instructed to focus entirely on the body and ignore the face. ERP data were recorded in the same way as in Part 1.

### Participants

The same 18 healthy right-handed Beijing Forestry University undergraduate students in Part 1 participated in Part 2 of the study. The order of Part 1 and 2 were counterbalanced across the subjects. Between the two parts the subjects took a short break and filled out a set of questionnaires as a filter task. Data from four participants were excluded because of excessive artifacts. Statistical analyses were then performed on the data from the remaining 14 participants, which included 7 females and 7 males. All participants had normal or corrected-to-normal vision, and had no history of affective disorder.

### Materials

The compound stimuli used in this part were the same as those in Part 1, but the red fixation point was marked differently on the center of the chest.

### Procedure

During EEG recordings, subjects were seated in the same room as in Part 1. The face-body compound stimuli were presented in a similar procedure. Each trial began with a 200 ms black fixation point, which was located in the upper 42% of the screen and was approximately the same height as the red fixation point on the center of the chest. Then, as in Part 1, after a blank screen randomized from 100 ms to 300 ms was presented, the stimuli were presented for 200 ms, followed by another black screen lasting for 1000 ms before the next trial. The participant's task was to judge the emotion expressed by the body as quickly and accurately as possible by pressing F or J on the keyboard. The key for happiness or fear was counterbalanced among the participants. All the compound stimuli were pseudo-randomized into four blocks as in Part 1, and each block contained 90 trials. The participants were asked to keep their eyes fixed on the body during the task. Before the real trials, all participants went through a training session to get familiar with the procedure.

### ERP Recording & Analysis

EEG was recorded and analyzed as in Part 1.

## Part 2 Results

### Behavioral Data

Two-way repeated measures ANOVAs of bodily expression (happiness *vs.* fear) by congruence (congruent *vs.* incongruent) were employed to analyze reaction times (RTs) and accuracy. A main effect of congruence was found for both accuracy [*F* (1,13) = 24.25, *p*<.001] and RTs [*F* (1,13) = 42.29, *p*<.001] in the task of judging bodily expressions. Participants made better (*M* = 91±4%) and faster (*M* = 542±87 ms) decisions when bodies were accompanied by faces with congruent expressions than when they were accompanied by faces with incongruent expressions (Accuracy: *M* = 85±5%; RT: *M* = 590±85 ms).

### ERP data

#### P1 component

**Figure 3:**
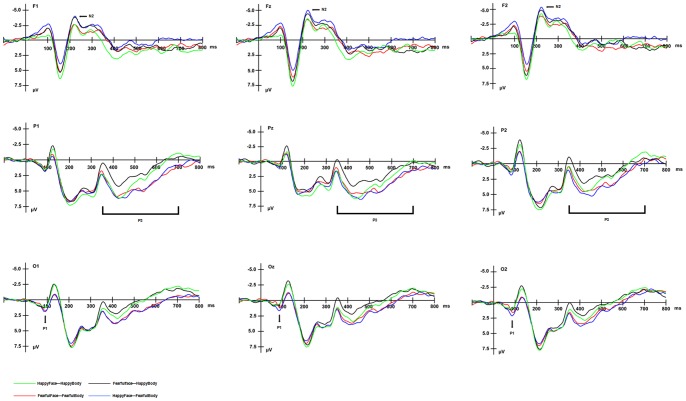
Group average ERPs for the four different compound stimuli at indicated electrode sites in Experiment2.

A main effect of bodily expression [*F* (1, 13) = 8.26, *p*<.01] was found for P1 amplitude at all three occipital sites (Figure 3). Larger P1 amplitudes were elicited by viewing compound images with fearful bodies (*M* = 1.74 µV) than by viewing compound images with happy bodies (*M* = 0.05 µV). Similar to Part 1, we again observed a main effect of electrode site [*F* (2, 26) = 6.69, *p*<.022], although post hoc tests did not reveal significant differences between the three electrode sites. There were no other significant effects.

A repeated-measures ANOVA with facial expression (happiness *vs.* fear), congruence (congruent *vs.* incongruent), and electrode site as factors pointed to a significant interaction of facial expression and congruence [*F* (1, 13) = 8.26, *p*<.01], although facial expression did not emerge as a significant factor on its own. Post hoc tests revealed that when the facial expression was fearful, larger P1s were elicited by congruent images than by incongruent images (*p*<.06). Conversely, when the facial expression was happy, larger P1s were elicited by incongruent images than by congruent images (*p*<.01). In addition, a main effect of electrode site [*F* (2, 26) = 6.69, *p*<.022] was again found, although post hoc tests did not reveal significant differences between the three electrode sites.

#### N2 component

As demonstrated in Figure 3, there was a main effect of congruence [*F* (1, 13) = 14.54, *p*<.001] for the amplitude of the N2 component. The compound stimuli with incongruent expressions elicited larger N2 (*M* = −4.24 µV) amplitudes than those with congruent expressions (*M* = −3.13 µV). No other main effects or interactions were found.

#### P3 component

As shown in Figure 3, a main effect of bodily expression for the mean amplitude of the P3 component was observed [*F* (1, 13) = 12.18, *p*<.001]. Compound images with fearful bodies (*M* = 3.60 µV) elicited more pronounced P3 waveforms than compound images with happy bodies (*M* = 2.19 µV). No other main effects or interactions were found.

## Part 2 Discussion

Part 2 of the study demonstrated the time course of integrating emotional signals from the face and body when participants were instructed explicitly to focus on the body. First, similar to Part 1, an early negativity bias towards threatening bodily expressions was found for the P1 component. Specifically, compound images with fearful bodies enhanced P1 amplitudes relative to compound images with happy bodies. No such effect on P1 amplitudes emerged when the participants viewed compound images with fearful versus happy faces. Second, detection of incongruence was captured by the N2 component, with larger N2 amplitudes elicited in the incongruent condition. Third, later in the evaluative processes, threatening signals from the body attracted sustained attention and received more elaboration, as evidenced by compound images with fearful bodies eliciting more pronounced P3 waveforms than compound images with happy bodies.

## General Discussion

Using different tasks to manipulate the focus of attention, the two parts of the present study have shown that the integration of emotional signals from the face and the body starts to occur during the early stage of information processing. As time unfolds, the integration of emotional signals demonstrates different features in different stages of processing. In addition, we have found that the focus of attention, on the face or on the body, modulates the integration pattern during later evaluative processes. These findings are important for several reasons, which are elaborated below.

### The hypothesis of three stages of integration of facial and bodily expressions

First, the findings have demonstrated distinct integrating features in different stages of information processing. The integration of facial and bodily expressions has previously been found to occur very early in the course of information processing [12]. However, in previous studies, the time course was not fully observed. The present findings indicate that the integration can be divided into three stages. Thus our understanding of the temporal dynamics of the integration of emotional signals from the face and body has been extended.

The first stage of integration is characterized by the automatic and rapid extraction of threatening information from the body, indexed by enhanced P1 amplitudes. These data support our proposed three-stage model of processing of facial expressions [24] in which we suggest that negative affect information is subjected to automatic processing during the earliest stage. Other researchers investigating the time course of processing emotional expressions have also found that negative emotional information is captured at very short latencies, within approximately 120 ms after stimulus onset [25], [26], [27], which is consistent with the finding in our study. Given that emotional signals from the body are extracted rapidly, even when they are not the focus of attention and task-irrelevant in Part 1, it is reasonable to infer that the processing of bodily expression may not require visual awareness. This lends support to the existence of a subcortical network, as hypothesized in de Gelder's model of body perception [2]. In addition to our findings, there has been other evidence for a subcortical network involved in body perception. For instance, in de Gelder and Hadjikhani's study, in which fearful, happy and neutral whole body images with the face blurred were presented to the blind field of a patient with unilateral striate cortex damage, emotional body images were recognized significantly above chance level and distinct patterns of brain activities were observed when fearful, happy and neutral body images were presented [5]. In another study by Tamietto and colleagues, patients with visual extinction and hemispatial neglect after right parietal injury were presented with fearful, happy, and neutral whole body images [28]. The results showed that, with simultaneous bilateral stimulation, fearful body images displayed in the contralesional left visual field with neutral body images in the ipsilesional right visual field were detected more often than neutral or happy body images displayed in the contralesional left visual field. Echoing our results, these two studies both suggest that the processing of bodily expressions may not require the involvement of the visual cortex. In addition, based on our findings that a larger P1 is elicited by compound images with fearful bodies than by compound images with happy bodies, regardless of whether attention is focused on the face or on the body, and that no such effect exists with fearful versus happy faces, it is reasonable to infer that there is a distinctive negativity bias towards threatening bodily expressions. These findings, therefore, extends our understanding of P1 as an index of early differentiation of positive and negative stimuli [29], which include body expressions. In addition, the findings also extend previous findings of negative bias in processing facial expressions [25], [27] to conditions when two types of emotional information are present.

The second stage of integration is characterized by the detection of any incongruent information between facial and bodily expressions. In both parts of the present study, N2 exhibited greater amplitudes when the emotional signals conveyed by the face and body were incongruent than when they were congruent. Kanske and Kotz [30] have proposed that there is an adaptive mechanism enabling rapid processing of conflict signaled by emotional stimuli, thus reducing the time that an organism needs to respond to potential threats, which may lead to a survival advantage. Lending support to this hypothesis, a recent study [31] found that conflict was prioritized in emotional, relative to neutral, situations, accompanied by a modulation of N2 amplitudes, which is consistent with the finding in our study. Another previous study [12], however, suggested that conflict between emotions conveyed by the face and body could be detected earlier than this second stage period and that the conflict detection was reflected by P1 amplitudes when participants were asked to judge facial expressions from face-body compound stimuli. The inconsistency between our findings and those of the previous study may be due to the differences in the participant's fixation point. In the previous study, the fixation point was at the chest level, whereas in Part 1 of our study, the fixation point was on the face, which led to greater accuracy in judging facial expression (M = 90%) relative to the previous study (M = 74%). Detection of incongruence between emotional signals from the face and the body first requires perception of emotional information from both sources. Given that setting the fixation point to the chest in the previous study led participants to fixate on the body, it is reasonable to infer that the participants would perceive emotional information from the body faster, and thus detect incongruence between emotional signals from the face and the body faster than in Part 1 of our study when participants fixated on the face. Thus, it may be that the fixation point set on the chest caused the incongruence to be captured earlier by P1 amplitudes in the previous study, whereas the detection of incongruence was delayed and captured by N2 amplitudes in our study.

In the third stage, further integration proceeds through more elaborate evaluation. Prior research has suggested that P3 may index sustained selective attention and be associated with late conflict resolution via top-down [32] and evaluative processing [20]. A recent study [33] found that incongruence between attended and unattended emotional face stimuli further enhanced P3 amplitudes after conflict detection in a Go/NoGo task. In contrast, the results of Part 1 in our study showed that compound face-body images with congruent emotional signals elicited more pronounced P3 waveforms, whereas the results of Part 2 showed that compound images with fearful bodies enhanced P3 waveforms after conflict detection in the second stage of processing. Taken together, these findings suggest that, in contrast to the way emotional signals from attended and unattended faces are integrated, integration of emotional signals from the attended face and unattended body is driven by evaluation of their congruent meaning rather than by detection of conflict, whereas integration of signals from an unattended face and attended body is driven by elaboration of threatening information from the body during this stage. Ito and colleagues [34] have previously proposed that a negative bias in emotional information processing occurs during evaluative processes. There has been some evidence for this hypothesis. A negative bias has been found to emerge when subjects are evaluating isolated emotional faces [24] and natural scenes [35]. Interestingly, a negative bias was also observed in our study when participants were evaluating emotional face-body images with their attention focused on the body, which extends our understanding of the conditions under which the negative bias may occur during the evaluation stage of emotional processing.

### The role of attention focus in integration of facial and bodily expressions

The most important implication of our findings pertains to the role of attention focus in modulating the integration pattern in the third stage of processing, when emotional signals from the face and body are both available. When attention is focused on the face, conflicts appear to be resolved in the third stage when congruent meaning of emotional signals integrated from the face and body seems to receive more sustained attention and more elaboration. In a previous fMRI study [13] in which participants were asked to judge facial expressions and ignore voices, more neural activity in the amygadala was found when facial expressions were paired with a congruent emotional voice relative to an incongruent voice. Similarly, we observed that when participants were instructed to judge facial expressions and ignore the body, congruent facial-bodily expression pairs elicited more neural activity than incongruent pairs, indexed by larger P3 amplitudes. On the other hand, when attention was focused on the body, no effect of congruent meaning was observed in P3. Instead, threat signals from the body appeared to be evaluated more elaborately. In another imaging study [36], greater neural activity was observed in the fusiform gyrus when subjects were judging bodily expressions from fearful face-body images with the face blurred compared to when they were evaluating happy or neutral body images. Similarly, we found greater neural activity revealed by larger P3s when subjects were judging bodily expressions from compound face-body images with fearful bodies than when they were evaluating images with happy bodies. The interaction of emotion and attention has been a topic of great interest in social cognition. Appraisal theories of emotion proposed by Scherer [37] and Sander and colleagues [38] have suggested that multiple levels of processing are involved in the evaluation of emotional information, with some processes happening relatively independent of attention, and others more dependent on attention and current tasks. There has been some evidence supporting this proposal. Researchers have found that emotional faces can be processed without visual awareness [39], [40], and the focus of attention can modulate both processing of emotional faces [41] and emotional voices [42], eliciting differential neural activities. Lending further support to this proposal and extending previous findings, our results indicate that attentional focus can also modulate the evaluation of emotional information when two types of emotional stimuli are present.

### Bodily expression dominance

An interesting question that remains is the cause of the difference in integration patterns observed in the two parts of the study. One possible cause that can be advanced for this difference is that emotional signals, especially negative ones from the face and body, may be disproportionately weighted. The results of our present study have provided some evidence for this notion. First, in the first stage of integration, a negativity bias towards bodily expressions instead of facial expressions is observed, whether the focus is on the face or body. Second, in the third stage of integration, threatening information from bodily expressions receives more elaboration when the focus is on the body, but no such effect is observed with facial expressions when the focus is on the face. In short, there might be a ‘bodily expression dominance’, leading to asymmetric influence of facial and bodily expressions when a person is processing emotional face-body stimuli. This phenomenon may resemble dominance of a particular sensory modality when there is conflicting information coming from different sensory modalities. In cross-modal studies [43], [44], [45], vision has been found to have an advantage over other senses in information processing; thus, the hypothesis of ‘visual dominance’ was proposed. The effect of ‘visual dominance’ has been investigated by manipulating the focus of attention. In a recent study, by using a switching attention paradigm to investigate the cross-modal interference between simultaneously presented visual and auditory stimuli, Lukas and colleagues [46] found that the presence of irrelevant auditory stimuli did not affect responses to visual stimuli as much as the presence of irrelevant visual stimuli affected responses to auditory stimuli, revealed by a stronger interference effect for auditory stimuli than for visual stimuli. The effect of ‘visual dominance’ was shown by an asymmetric influence of visual and auditory stimuli, which is similar to the way facial and bodily expressions affected each other in our study. Our hypothesis of ‘bodily expression dominance’ also fits well with some findings in fMRI studies. For instance, van de Riet, Grezes and de Gelder [36] found that viewing static emotional bodies triggered more activity in the left and right fusiform gyrus than viewing emotional faces. Using dynamic face and body stimuli, another recent study by Kret and colleagues [47] also showed that emotional bodies triggered more activity than emotional faces in a number of brain areas, which included the fusiform gyrus, extrastriate body area, temporoparietal junction, superior parietal lobule, primary somatosensory cortex and the thalamus.

A possible alternative explanation is that the ‘bodily expression dominance’ is an artifact of the fact that the body is larger than the face in the image, and thus, perhaps, difficult to ignore. Given that the presentation time of the compound stimuli was very short, this alternative explanation would predict that the accuracy of judging facial expressions should be relatively poor when the participant is asked to focus on the face and to ignore the body. However, this prediction is not supported by the result of Part 1, in which subjects showed good accuracy in judging emotions based on facial expression. Moreover, because the face and the body are normally seen together in their natural proportions, keeping those natural differences in size should maintain the ecological validity of the assessment of competition between emotional signals from the face and the body. A more direct investigation of the theory of disproportionate weighting should be addressed in future research. In addition, the mechanism by which bodily expressions could receive this disproportionate weighting is also an interesting question to explore in the future.

In conclusion, the current findings expand our understanding of the integration of emotional signals from the face and the body. Our findings fit well with the hypothesis that integration occurs in three stages. A more pronounced negativity bias towards bodily expressions relative to facial expressions emerges in the first stage. Detection of incongruence, if any, then occurs in the second stage, followed by a merging of emotional information by elaborate evaluation in the third stage. The focus of one's attention appears to play a role in modulating integration patterns during the third stage.

## Supporting Information

File S1 **A copy of consent form for publication of** **Figure 1** **from the participant concerned.**
(PDF)

File S2 **A copy of permission form for publication of** **Figure 1** **from the copyright holder of it.**
(PDF)

## References

[1] de Gelder B, Van den Stock J, Meeren HK, Sinke CB, Kret ME, et al (2010) Standing up for the body. Recent progress in uncovering the networks involved in the perception of bodies and bodily expressions. Neurosci Biobehav Rev 34: 513–527. 19857515 10.1016/j.neubiorev.2009.10.008

[2] de Gelder B (2006) Towards the neurobiology of emotional body language. Nat Rev Neurosci 7: 242–249. 16495945 10.1038/nrn1872

[3] van Heijnsbergen CC, Meeren HK, Grezes J, de Gelder B (2007) Rapid detection of fear in body expressions, an ERP study. Brain Res 1186: 233–241. 17996856 10.1016/j.brainres.2007.09.093

[4] Hadjikhani N, de Gelder B (2003) Seeing fearful body expressions activates the fusiform cortex and amygdala. Curr Biol 13: 2201–2205. 14680638 10.1016/j.cub.2003.11.049

[5] de Gelder B, Hadjikhani N (2006) Non-conscious recognition of emotional body language. Neuroreport 17: 583–586. 16603916 10.1097/00001756-200604240-00006

[6] Vuilleumier P, Righart R (2011) Attention and automaticity in processing facial expressions. In: Calder AJ, Rhodes G, Johnson MH, Haxby JV, editors. Oxford handbook of face perception. Oxford: Oxford University Press. pp. 449–534.

[7] de Gelder B, Bocker KB, Tuomainen J, Hensen M, Vroomen J (1999) The combined perception of emotion from voice and face: early interaction revealed by human electric brain responses. Neurosci Lett 260: 133–136. 10025717 10.1016/s0304-3940(98)00963-x

[8] Pourtois G, de Gelder B, Vroomen J, Rossion B, Crommelinck M (2000) The time-course of intermodal binding between seeing and hearing affective information. Neuroreport 11: 1329–1333. 10817616 10.1097/00001756-200004270-00036

[9] Pourtois G, Debatisse D, Despland PA, de Gelder B (2002) Facial expressions modulate the time course of long latency auditory brain potentials. Brain Res Cogn Brain Res 14: 99–105. 12063133 10.1016/s0926-6410(02)00064-2

[10] Jessen S, Kotz SA (2011) The temporal dynamics of processing emotions from vocal, facial, and bodily expressions. Neuroimage 58: 665–674. 21718792 10.1016/j.neuroimage.2011.06.035

[11] Van den Stock J, Righart R, de Gelder B (2007) Body expressions influence recognition of emotions in the face and voice. Emotion 7: 487–494. 17683205 10.1037/1528-3542.7.3.487

[12] Meeren HK, van Heijnsbergen CC, de Gelder B (2005) Rapid perceptual integration of facial expression and emotional body language. Proc Natl Acad Sci U S A 102: 16518–16523. 16260734 10.1073/pnas.0507650102 PMC1283446

[13] Dolan RJ, Morris JS, de Gelder B (2001) Crossmodal binding of fear in voice and face. Proc Natl Acad Sci U S A 98: 10006–10010. 11493699 10.1073/pnas.171288598 PMC55568

[14] Ekman P, Friesen WV (1974) Detecting deception from body or face. J Pers Soc Psychol 29: 288–298.

[15] Stienen BM, Tanaka A, de Gelder B (2011) Emotional voice and emotional body postures influence each other independently of visual awareness. PLoS One 6: e25517. 22003396 10.1371/journal.pone.0025517 PMC3189200

[16] Talsma D, Doty TJ, Woldorff MG (2007) Selective attention and audiovisual integration: is attending to both modalities a prerequisite for early integration? Cereb Cortex 17: 679–690. 16707740 10.1093/cercor/bhk016

[17] Clark VP, Hillyard SA (1996) Spatial selective attention affects early extrastriate but not striate components of the visual evoked potential. J Cogn Neurosci 8: 387–402. 23961943 10.1162/jocn.1996.8.5.387

[18] Liotti M, Woldorff MG, Perez R, Mayberg HS (2000) An ERP study of the temporal course of the Stroop color-word interference effect. Neuropsychologia 38: 701–711. 10689046 10.1016/s0028-3932(99)00106-2

[19] Nieuwenhuis S, Yeung N, van den Wildenberg W, Ridderinkhof KR (2003) Electrophysiological correlates of anterior cingulate function in a go/no-go task: effects of response conflict and trial type frequency. Cogn Affect Behav Neurosci 3: 17–26. 12822595 10.3758/cabn.3.1.17

[20] Willadsen-Jensen EC, Ito TA (2006) Ambiguity and the timecourse of racial perception. Soc Cogn 24: 580–606.

[21] Gong X, Huang YX, Wang Y, Luo YJ (2011) Revision of the Chinese facial affective picture system. Chin Ment Health J 25: 40–46.

[22] Luck SJ (2005) An introduction to the event-related potential technique. Cambridge MA: MIT press.

[23] Semlitsch HV, Anderer P, Schuster P, Presslich O (1986) A solution for reliable and valid reduction of ocular artifacts, applied to the P300 ERP. Psychophysiology 23: 695–703. 3823345 10.1111/j.1469-8986.1986.tb00696.x

[24] Luo W, Feng W, He W, Wang NY, Luo YJ (2010) Three stages of facial expression processing: ERP study with rapid serial visual presentation. Neuroimage 49: 1857–1867. 19770052 10.1016/j.neuroimage.2009.09.018 PMC3794431

[25] Ashley V, Vuilleumier P, Swick D (2004) Time course and specificity of event-related potentials to emotional expressions. Neuroreport 15: 211–216. 15106860 10.1097/00001756-200401190-00041

[26] Vuilleumier P, Pourtois G (2007) Distributed and interactive brain mechanisms during emotion face perception: evidence from functional neuroimaging. Neuropsychologia 45: 174–194. 16854439 10.1016/j.neuropsychologia.2006.06.003

[27] Eimer M, Holmes A (2002) An ERP study on the time course of emotional face processing. Neuroreport 13: 427–431. 11930154 10.1097/00001756-200203250-00013

[28] Tamietto M, Geminiani G, Genero R, de Gelder B (2007) Seeing fearful body language overcomes attentional deficits in patients with neglect. J Cogn Neurosci 19: 445–454. 17335393 10.1162/jocn.2007.19.3.445

[29] Smith NK, Cacioppo JT, Larsen JT, Chartrand TL (2003) May I have your attention, please: electrocortical responses to positive and negative stimuli. Neuropsychologia 41: 171–183. 12459215 10.1016/s0028-3932(02)00147-1

[30] Kanske P, Kotz SA (2011) Emotion triggers executive attention: anterior cingulate cortex and amygdala responses to emotional words in a conflict task. Hum Brain Mapp 32: 198–208. 20715084 10.1002/hbm.21012 PMC6870409

[31] Kanske P, Kotz SA (2010) Modulation of early conflict processing: N200 responses to emotional words in a flanker task. Neuropsychologia 48: 3661–3664. 20654636 10.1016/j.neuropsychologia.2010.07.021

[32] Nieuwenhuis S, Aston-Jones G, Cohen JD (2005) Decision making, the P3, and the locus coeruleus-norepinephrine system. Psychol Bull 131: 510–532. 16060800 10.1037/0033-2909.131.4.510

[33] Wang L, Fu S, Feng C, Luo W, Zhu X, et al (2012) The neural processing of fearful faces without attention and consciousness: An event-related potential study. Neurosci Lett 506: 317–321. 22155093 10.1016/j.neulet.2011.11.034

[34] Ito TA, Larsen JT, Smith NK, Cacioppo JT (1998) Negative information weighs more heavily on the brain: the negativity bias in evaluative categorizations. J Pers Soc Psychol 75: 887–900. 9825526 10.1037//0022-3514.75.4.887

[35] Huang YX, Luo YJ (2006) Temporal course of emotional negativity bias: an ERP study. Neurosci Lett 398: 91–96. 16446031 10.1016/j.neulet.2005.12.074

[36] van de Riet WA, Grezes J, de Gelder B (2009) Specific and common brain regions involved in the perception of faces and bodies and the representation of their emotional expressions. Soc Neurosci 4: 101–120. 19255912 10.1080/17470910701865367

[37] Scherer KR (2001) Appraisal considered as a process of multilevel sequential checking. In: Scherer KR, Schorr A, Johnstone T, editors. Appraisal Processes in Emotion: Theory, Methods, Research. New York and Oxford: Oxford University Press. pp. 92–120.

[38] Sander D, Grandjean D, Scherer KR (2005) A systems approach to appraisal mechanisms in emotion. Neural Netw 18: 317–352. 15936172 10.1016/j.neunet.2005.03.001

[39] Vuilleumier P, Schwartz S (2001) Beware and be aware: capture of attention by fear-relevant stimuli in patients with unilateral neglect. NeuroReport 12: 1119–1122. 11338176 10.1097/00001756-200105080-00014

[40] Vuilleumier P, Armony JL, Clarke K, Husain M, Driver J, et al (2002) Neural response to emotional faces with and without awareness: event-related fMRI in a parietal patient with visual extinction and spatial neglect. Neuropsychologia 40: 2156–2166. 12208011 10.1016/s0028-3932(02)00045-3

[41] Holmes A, Vuilleumier P, Eimer M (2003) The processing of emotional facial expression is gated by spatial attention: evidence from event-related brain potentials. Brain Res Cogn Brain Res 16: 174–184. 12668225 10.1016/s0926-6410(02)00268-9

[42] Sander D, Grandjean D, Pourtois G, Schwartz S, Seghier ML, et al (2005) Emotion and attention interactions in social cognition: brain regions involved in processing anger prosody. Neuroimage 28: 848–858. 16055351 10.1016/j.neuroimage.2005.06.023

[43] Collignon O, Girard S, Gosselin F, Roy S, Saint-Amour D, et al (2008) Audio-visual integration of emotion expression. Brain Res 1242: 126–135. 18495094 10.1016/j.brainres.2008.04.023

[44] Hartcher-O'Brien J, Levitan C, Spence C (2010) Extending visual dominance over touch for input off the body. Brain Res 1362: 48–55. 20850418 10.1016/j.brainres.2010.09.036

[45] Bertelson P, Radeau M (1981) Cross-modal bias and perceptual fusion with auditory-visual spatial discordance. Percept Psychophys 29: 578–584. 7279586 10.3758/bf03207374

[46] Lukas S, Philipp AM, Koch I (2010) Switching attention between modalities: further evidence for visual dominance. Psychol Res 74: 255–267. 19517132 10.1007/s00426-009-0246-y

[47] Kret ME, Pichon S, Grezes J, de Gelder B (2011) Similarities and differences in perceiving threat from dynamic faces and bodies. An fMRI study. Neuroimage 54: 1755–1762. 20723605 10.1016/j.neuroimage.2010.08.012

