# Supplementary material for: Do Bodily Expressions Compete with Facial Expressions? Time Course of Integration of Emotional Signals from the Face and the Body
Source: PLoS One. 2013 Jul 23;8(7):e66762. doi: 10.1371/journal.pone.0066762 (PMC3720771; doi:10.1371/journal.pone.0066762)
Supplement: File S1 — A copy of consent form for publication of Figure 1 from the participant concerned. (PDF) [file pone.0066762.s001.pdf]

Informed Consent—Student Research

[A Picture System of Bodily Expressions]
[Yue-jia Luo]

I am a graduate student in the School of Brain and Cognitive Sciences at Beijing Normal University. I am here to conduct a study that will take photos of bodily expressions for setting up a local picture database.

Before we begin, I would like to take a minute to explain why I am inviting you to participate and what I will be doing with the information you provide to me. Please stop me at any time if you have any questions. After I've told you a bit more about my project, you can decide whether or not you would like to participate.

I am doing this research as part of my studies in the School of Brain and Cognitive Sciences at Beijing Normal University. I will be taking photos of you while you express emotions with your whole body and will use these photos to set up a local database of bodily expression. I may also use these photos in articles that might be published, as well as in academic presentations.

Participation should take about 5 minutes. Participation is on a purely voluntary basis. You will be asked to express six different emotions with your whole body, which include happiness, sadness, fear, surprise, anger and disgust. There would be minimal risks to complete the task and your data would be confidential and your personal information would never be released to the public. Your photos that will be used in my thesis, articles or presentations will be coded and there will be no identifying information about you. By participating in the study, you would be contributing to setting up a local picture database.

If at any time and for any reason, you would prefer not to participate, please feel free not to. If at any time you would like to stop participating, please tell me. We can take a break, stop and continue at a later date, or stop altogether. You will not be penalized in any way for deciding to stop participation at any time.

You will get cash with the amount of RMB50.

If you have questions, you are free to ask them now. If you have questions later, you may contact me at [Yuanyuan Gu, School of Brain and Cognitive Sciences, Beijing Normal University, Beijing 100875, China, 13522882263, guyuanyuan15@yahoo.com.cn].

If you have any questions about your rights as a participant in this research, you can contact the following office at Beijing Normal University:

Social & Behavioral Sciences Institutional Review Board
Beijng Normal Universtiy
19 Xinjiekouwai Street
Beijing, China
Phone: (86) 10-5880-5456
Email: duboqi@bnu.edu.cn

Are you interested in participating in this study? If yes, please sign your name AND DATE here below

SIGNATURE OF SUBJECT: [illegible]

DATE: 06/05/2007
