# Supplementary material for: Do Bodily Expressions Compete with Facial Expressions? Time Course of Integration of Emotional Signals from the Face and the Body
Source: PLoS One. 2013 Jul 23;8(7):e66762. doi: 10.1371/journal.pone.0066762 (PMC3720771; doi:10.1371/journal.pone.0066762)
Supplement: File S2 — A copy of permission form for publication of Figure 1 from the copyright holder of it. (PDF) [file pone.0066762.s002.pdf]

Permission Form

The copyright of the material contained in Figure 1 belongs to the State Key Lab of Cognitive Neuroscience and Learning, Beijng Normal University. The material in Figure 1 has been permitted to use in the manuscript entitled "How Do Bodily Expressions Compete with Facial Expressions: The Time Course of Integrating Emotional Signals from the Face and the Body" and to publish in an open access journal.

北京师范大学认知神经科学与学习国家重点实验室
The State Key Lab of Cognitive Neuroscience and Learning, BNU
